# RNA Interference of TorsinA in *Acyrthosiphon pisum*

**Published:** 2026-05-20

**Authors:** Braden Allphin, James R Balthazor

**Affiliations:** Department of Chemistry, Fort Hays State University, Hays, United States of America

**Keywords:** RNAi, Knockdown, Pest mitigation, TOR1A

## Abstract

RNA interference (RNAi) represents a promising strategy for species-specific pest control by selectively silencing essential genes. The pea aphid, *Acyrthosiphon pisum*, is a major agricultural pest that reduces legume crop productivity and transmits plant pathogens, while current control methods rely heavily on insecticides with environmental and ecological drawbacks. In this study, the gene TOR1A, which encodes the AAA+ ATPase TorsinA involved in protein folding and endoplasmic reticulum-associated protein quality control, was evaluated as a target for RNAi-mediated mortality. Double-stranded RNA (dsRNA) targeting TOR1A was synthesized and delivered to adult aphids *via* an artificial feeding assay at concentrations of 0.1 μg/μL and 1.0 μg/μL. Survival was monitored at 6-hour intervals and analyzed using Kaplan meier survival curves with statistical comparisons performed using the mantel cox (log-rank) test.

Both dsRNA treatment groups exhibited significantly reduced survival relative to untreated controls, with a clear dose-dependent effect. The 0.1 μg/μL treatment produced a modest but significant increase in mortality (p=0.0136), whereas the 1.0 μg/μL treatment resulted in a markedly accelerated mortality rate (p=3.23 × 10^−14^). Direct comparison between treatment groups confirmed a significant difference in survival (p=2.28 × 10^−8^). Hazard ratio analysis further supported these findings, with the 0.1 μg/μL treatment yielding a Hazard Ratio (HR) of 1.85 (95% CI:1.14–3.01) and the 1.0 μg/μL treatment yielding an HR of 8.46 (95% CI: 4.87–14.68) relative to control, while comparison between treatments produced an HR of 4.31 (95% CI: 2.58–7.20).

These results demonstrate that disruption of TOR1A significantly impairs survival in *A. pisum*, likely through accumulation of misfolded proteins, induction of endoplasmic reticulum stress and activation of apoptotic pathways. The strong dose-dependent response highlights TOR1A as a robust and effective RNAi target. This study provides further evidence that targeting protein homeostasis pathways is a viable and selective approach for aphid control and supports the development of RNAi-based strategies as alternatives to conventional insecticides.

## INTRODUCTION

The pea aphid, *Acyrthosiphon pisum*, is a sap-feeding pest of legume crops and can reduce plant vigor, diminish yield and contribute to disease spread [1,2]. Although insecticides remain a common control strategy, their use can adversely affect non-target organisms and the surrounding environment [3]. Ribonucleic Acid (RNA) interference (RNAi) provides an attractive alternative because it can be directed toward species-relevant molecular targets [4,5].

This study focused on TOR1A, which encodes TorsinA, a protein involved in protein folding, stabilization and handling of misfolded proteins [6–9]. Disruption of these processes is expected to impair protein homeostasis, increase cellular stress and ultimately reduce organismal survival [10,11]. We hypothesized that delivery of TOR1A dsRNA to adult aphids would increase mortality relative to untreated controls and that the higher dsRNA concentration would produce a stronger effect.

## MATERIALS AND METHODS

Total RNA was isolated from adult *A. pisum* using TRIzol reagent, followed by chloroform extraction and isopropanol precipitation. Complementary Deoxyribonucleic Acid (cDNA) was synthesized from the RNA template and used for dsRNA production with gene-specific primers targeting TOR1A. The dsRNA product was verified and quantified before use in feeding studies.

For feeding assays, 50 adult aphids were placed in each treatment group and offered an artificial diet enclosed between two layers of parafilm. Treatments consisted of control diet without dsRNA, diet containing approximately 0.1 μg/μL TOR1A dsRNA and diet containing approximately 1.0 μg/μL TOR1A dsRNA. Mortality was recorded every 6 h until all aphids had died.

Hazard Ratios (HR) with 95% Confidence Intervals (CI) were calculated for each comparison. The 0.1 μg/μL treatment exhibited an HR of 1.85 (95% CI:1.14–3.01) relative to control, while the 1.0 μg/μL treatment exhibited an HR of 8.46 (95% CI:4.87–14.68) relative to control. Comparison between treatment groups yielded an HR of 4.31 (95% CI:2.58–7.20).

## RESULTS AND DISCUSSION

Treatment with TOR1A dsRNA reduced survival relative to the untreated control group (Figure 1). The effect was concentration dependent. Aphids exposed to 1.0 μg/μL TOR1A dsRNA showed the fastest decline in survival, whereas aphids exposed to 0.1 μg/μL TOR1A dsRNA died more rapidly than the control group but more slowly than the 1.0 μg/μL group.

Hazard Ratios (HR) with 95% Confidence Intervals (CI) were calculated for each comparison. The 0.1 μg/μL treatment exhibited an HR of 1.85 (95% CI:1.14–3.01) relative to control, while the 1.0 μg/μL treatment exhibited an HR of 8.46 (95% CI:4.87–14.68) relative to control. Comparison between treatment groups yielded an HR of 4.31 (95% CI:2.58–7.20).

These findings are consistent with the hypothesis that interference with TOR1A disrupts protein homeostasis in *A. pisum*. Because TorsinA is associated with protein folding and management of misfolded proteins, reduced TOR1A activity could be expected to increase cellular stress and compromise viability. Together, the survival curves and statistical analysis identify TOR1A as a promising RNAi target for pea aphid control [12–18].

Hazard Ratios (HR) with 95% Confidence Intervals (CI) were calculated for each comparison. The 0.1 μg/μL treatment exhibited an HR of 1.85 (95% CI:1.14–3.01) relative to control, while the 1.0 μg/μL treatment exhibited an HR of 8.46 (95% CI:4.87–14.68) relative to control. Comparison between treatment groups yielded an HR of 4.31 (95% CI:2.58–7.20) as shown in Figure 1.

## CONCLUSION

This study demonstrates that RNA interference targeting TOR1A significantly reduces survival in *Acyrthosiphon pisum* in a clear dose-dependent manner, supporting the hypothesis that disruption of protein homeostasis is a viable mechanism for inducing aphid mortality. These findings directly align with the established biological role of TorsinA as an AAA+ ATPase involved in protein folding, Endoplasmic Reticulum (ER) function and maintenance of proteostasis. Disruption of this pathway is expected to impair the cell’s ability to properly fold and process proteins, resulting in accumulation of misfolded proteins and activation of ER stress pathways, including the Unfolded Protein Response (UPR).

The observed increase in mortality following TOR1A knockdown is consistent with prolonged ER stress leading to activation of apoptotic signaling cascades. Given the central role of proteostasis in maintaining cellular viability, particularly in metabolically active and rapidly reproducing organisms such as aphids, interference with this pathway represents a strategic vulnerability. These results are further supported by previous studies demonstrating that RNAi targeting components of the UPR and protein-folding machinery in *A. pisum* leads to increased mortality and reduced fitness.

Importantly, the strong dose-dependent response observed in this study highlights the necessity of achieving sufficient dsRNA uptake to surpass a threshold level of gene silencing. This is consistent with prior work indicating that RNAi efficiency in insects is influenced by dsRNA stability, uptake and degradation mechanisms. The significant hazard ratios and survival differences observed at higher concentrations suggest that TOR1A is a robust and sensitive RNAi target capable of producing biologically meaningful effects when adequately delivered.

From an applied perspective, these findings support the broader concept introduced in the Introduction: That species-specific RNAi strategies can provide an environmentally sustainable alternative to traditional insecticides. Targeting essential genes such as TOR1A allows for selective disruption of pest populations while minimizing off-target effects on beneficial organisms. Furthermore, the potential integration of dsRNA expression into transgenic crops represents a promising avenue for long-term pest management.

Future work should focus on validating the extent of TOR1A knockdown at the transcript and protein levels using quantitative PCR and proteomic approaches, as well as assessing sublethal effects such as fecundity and developmental timing. Additionally, combining TOR1A targeting with other genes involved in proteostasis or stress response pathways may enhance efficacy and reduce the likelihood of resistance development.

Overall, this study reinforces the critical role of protein homeostasis in aphid survival and establishes TOR1A as a compelling molecular target for RNAi-based pest control strategies. These findings contribute to the growing body of evidence supporting RNAi as a precise, scalable and environmentally responsible tool for agricultural pest management.

## Figures and Tables

**Figure 1:**
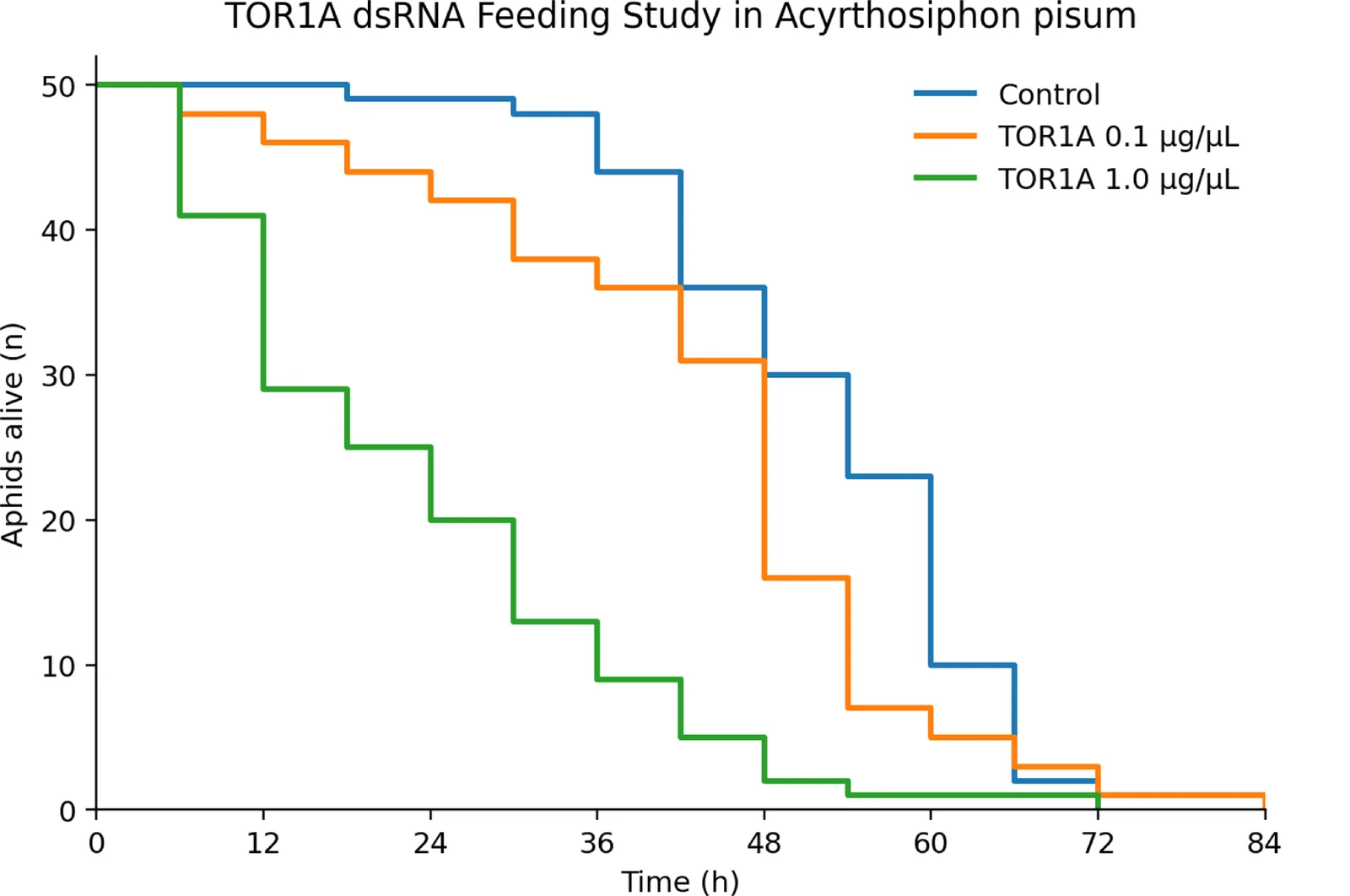
TOR1A dsRNA feeding study in *Acyrthosiphon pisum*.

## References

[1] The International Aphid Genomics Consortium. (2010) Genome sequence of the pea aphid *Acyrthosiphon pisum*. PLoS Biol. 8(2):e1000313. 10.1371/journal.pbio.1000313 PMC2826372 20186266

[2] McCornack B, Zukoff S, Whitworth RJ, Michaud JP, Schwarting HN, (2017) Alfalfa insect management 2017; Kansas State University: Manhattan KS. 2017.

[3] Tudi M, Ruan HD, Wang L, Lyu J, Sadler R, (2021) Agriculture development, pesticide application and its impact on the environment. Int J Environ Res Public Health. 18(3):1112. 10.3390/ijerph18031112 PMC7908628 33513796

[4] Whyard S, Singh AD, Wong S. (2009) *In vivo* knockdown of gene expression in insects using RNA interference. Insect Mol Biol. 18(4):401–408.

[5] Cooper AMW, Silver K, Zhang J, Park Y, Zhu KY, (2019) Molecular mechanisms influencing efficiency of RNA interference in insects. Pest Manag Sci. 75(1):18–28. 10.1002/ps.5126 29931761

[6] Hetz C (2012) The unfolded protein response: Controlling cell fate decisions under ER stress and beyond. Nat Rev Mol Cell Biol. 18;13:89–102. 10.1038/nrm3270 22251901

[7] Ron D, Walter P. (2007) Signal integration in the endoplasmic reticulum unfolded protein response. Nat Rev Mol Cell Biol. 8(7):519–529. 10.1038/nrm2199 17565364

[8] Yoshida H, Matsui T, Yamamoto A, Okada T, Mori K, (2001) XBP1 mRNA Is induced by ATF6 and spliced by IRE1 in response to ER stress to produce a highly active transcription factor. Cell. 28;107(7):881–891. 10.1016/s0092-8674(01)00611-0 11779464

[9] Calfon M, Zeng H, Urano F, Till JH, Hubbard SR, (2002) IRE1 couples endoplasmic reticulum load to secretory capacity by processing the XBP-1 mRNA. Nature. 415(6867):92–96. 10.1038/415092a 11780124

[10] Tabas I, Ron D. (2011) Integrating the mechanisms of apoptosis induced by endoplasmic reticulum stress. Nat Cell Biol. 13(3):184–190. 10.1038/ncb0311-184 PMC3107571 21364565

[11] Read A, Schröder M. (2021) The unfolded protein response: An overview. Biology (Basel). 10(5):384. 10.3390/biology10050384 PMC8146082 33946669

[12] Christiaens O, Swevers L, Smagghe G. (2014) dsRNA degradation in the pea aphid (Acyrthosiphon pisum) associated with lack of response in RNAi feeding and injection assay. Peptides. 53:307–314. 10.1016/j.peptides.2013.12.014 24394433

[13] Mao J, Zeng F. (2012) Feeding-based RNA interference of a gap gene is lethal to the pea aphid, *Acyrthosiphon pisum*. PLoS One. 7(11):e48718. 10.1371/journal.pone.0048718 PMC3492414 23144942

[14] Jaubert-Possamai S, Le Trionnaire G, Bonhomme J, Rispe C, Tagu D, (2007) Gene knockdown by RNAi in the pea aphid *Acyrthosiphon pisum*. BMC Biotechnol. 7:63. 10.1186/1472-6750-7-63 PMC2075495 17903251

[15] Mutti NS, Park Y, Reese JC, Reeck GR. (2006) RNAi knockdown of a salivary transcript leading to lethality in the pea aphid, *Acyrthosiphon pisum*. J Insect Sci. 6:38. 10.1673/031.006.3801 PMC2990334 20233093

[16] Ridder J, Balthazor J. (2022) RNA interference of three genes of the unfolded protein response: Activating transcription factor 4, eukaryotic translation initiation factor 2-alpha kinase and inositol-requiring enzyme 1 in *Acyrthosiphon pisum*. Biochem Mol Biol J.8:83. PMC13132143 42079322

[17] Davies G, Balthazor J. (2026) Evaluating the effect of rna interference of two heat shock proteins (HSPA1L, HSP90B1) in inducing apoptosis in Acyrthosiphon pis*um*. Biochem Mol Biol J. 12. PMC13108536 42039770

[18] Wessel EM, Tomich JM, Todd RB. (2019) Biodegradable drug-delivery peptide nanocapsules. ACS Omega. 4(18):20059–20063. 10.1021/acsomega.9b03245 PMC6882115 31788640

